# Brownian Dynamics Simulation of Microscale Thermophoresis in Liquid

**DOI:** 10.1021/acsomega.4c08170

**Published:** 2025-01-30

**Authors:** Koki Ide, Tetsuro Tsuji, Takayuki Suzuki, Kenji Setoura

**Affiliations:** † Advanced Course of Mechanical System Engineering, Kobe City College of Technology, Kobe, Hyogo 651-2194, Japan; ‡ Graduate School of Informatics, Kyoto University, Kyoto 606-8501, Japan; § Department of Mechanical Engineering, Kobe City College of Technology, Kobe, Hyogo 651-2194, Japan; ∥ Department of Electrical Materials and Engineering, Graduate School of Engineering, University of Hyogo, Himeji, Hyogo 671-2280, Japan

## Abstract

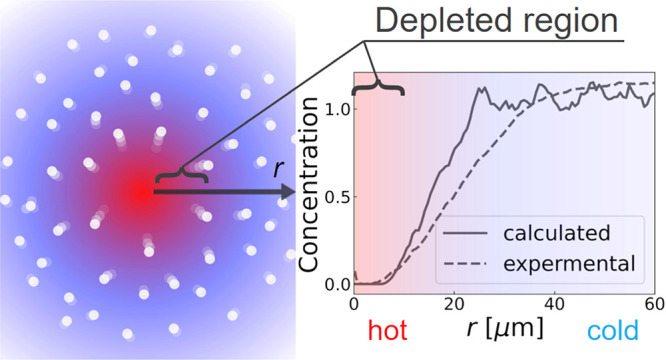

Microscale thermophoresis (MST) has garnered significant attention as a manipulation method for chemical species ranging from nanometers to micrometers in liquids. In particular, techniques for manipulating single nanometer-sized objects have been developed by driving MST through laser heating with near-infrared wavelengths focused down to submicron scales or via photothermal conversion of plasmonic nanoparticles. While MST simulations on a macroscopic scale can be addressed by solving the diffusion equation using the finite element method, alternative computational approaches are required to investigate thermophoretic behavior at the single-particle level. For this purpose, we have developed a numerical method for the thermophoresis of individual nanoparticles diffusing in a liquid by combining the finite element method for steady-state heat conduction with Brownian dynamics simulations. The scripts for the finite element method and Brownian dynamics calculations used in the present simulations are uploaded in the Supporting Information and freely available. The numerical results demonstrated satisfactory agreement with the experimental results of laser-induced thermophoresis performed on polystyrene nanoparticles with a diameter of 500 nm in water. This computational method is highly useful for controlling MST at the single-particle level, enabling the design of spatial temperature distributions and the evaluation of thermophoretic forces acting on individual nanoparticles.

## 1 Introduction

When tiny objects are exposed to steep temperature gradients in fluids, they may move along the temperature gradient. This temperature-induced migration is called *thermophoresis*.^1,2^ In general, objects move from the hot side to the cold side, but in some cases, movement in the opposite direction has been observed:^3^ the former is called “thermophobic motion” and the latter “thermophilic motion.” Although the thermophoresis, sometimes referred to as the Soret effect or thermodiffusion, was discovered in the 19th century, the past two decades have witnessed a surge of interest in microscale thermophoresis (MST) in liquids. This resurgence can be attributed to the pioneering work of Braun et al., who demonstrated the manipulation of DNA by inducing thermophoretic motion through the localized heating of an aqueous solution with a focused near-infrared laser.^4^ In principle, a temperature gradient of 10^5^–10^6^ K m^–1^ is required to induce the thermophoresis. By focusing laser light with an objective lens, a heating spot with a diameter of approximately 1 μm can be achieved,^5−7^ readily providing the necessary temperature gradient. A wide range of entities, including DNA,^4^ polystyrene and silica microparticles,^8^ plasmonic nanoparticles,^6^ Janus nanoparticles,^9−11^ surfactant micelles,^12^ polymers such as polyethylene glycol,^13^ vesicles,^14^ and *E. coli* bacteria,^15^ have been manipulated using MST in liquids.

In particular, there has been an increasing number of reports on methods for manipulating individual materials on the nano- to micrometer scale using MST, either by precisely designing spatial temperature distributions through the photothermal conversion of metal nanostructures,^12,16−18^ or by utilizing optical manipulation (optical tweezers) techniques in the past decade.^6,7,11,19−21^ In the case of MST at the single-particle level, it is difficult to simulate the thermophoretic behavior of individual particles using computational methods that solve the diffusion equation via finite element analysis, as previously reported.^22^ Therefore, to design optimal spatial temperature distributions for manipulating single nanoparticles and to evaluate the thermophoretic forces acting on individual particles, we have developed a Brownian dynamics simulation (BDS) model for MST in this article. The implementation of thermophoretic forces into the BDS was carried out through two steps: (i) calculating the temperature field using the finite element method (FEM), and (ii) computing the overdamped-Langevin equation using the finite-difference method. In step (i), a commercial FEM solver, COMSOL Multiphysics (version 6.2) was used. In step (ii), based on the temperature profile obtained in the FEM calculations on heat conduction, the thermophoretic force was incorporated into the overdamped-Langevin equation to numerically determine the particle motion.

As a representative specimen of the MST in liquid, we performed the above simulations for polystyrene nanoparticles (PSNPs) with a diameter of 500 nm in water. As a result, the NPs moved away from the heating spot over time. To verify the computational model, experiments were performed using equivalent samples and heating conditions, and satisfactory agreement was obtained between the experimental and numerical results in the behavior of thermophoretic depletion from the heating spot; this demonstrated that it is possible to quantitatively evaluate the thermophoretic force acting on individual nanoparticles. Because all the files for numerical calculations used in this article, including COMSOL files and Python codes for the BDS, were uploaded in Supporting Information, anyone can perform these simulations (see Supporting Information S1). This computational method enables the design of optimal temperature fields and the evaluation of thermophoretic forces acting on individual nanoparticles, thereby facilitating the development of more advanced MST manipulation techniques for single nanomaterials.

## 2 Numerical Simulations

The technical core in this study is the implementation of thermophoresis into the BDS. But, in any case, calculations of the thermophoretic forces necessitate the spatial distribution of temperature and temperature gradients. This section, therefore, will first describe the heat conduction calculations, and subsequently outline the BDSs incorporating thermophoretic effects.

### 2.1 Steady-State Heat Conduction

**Figure 1:**
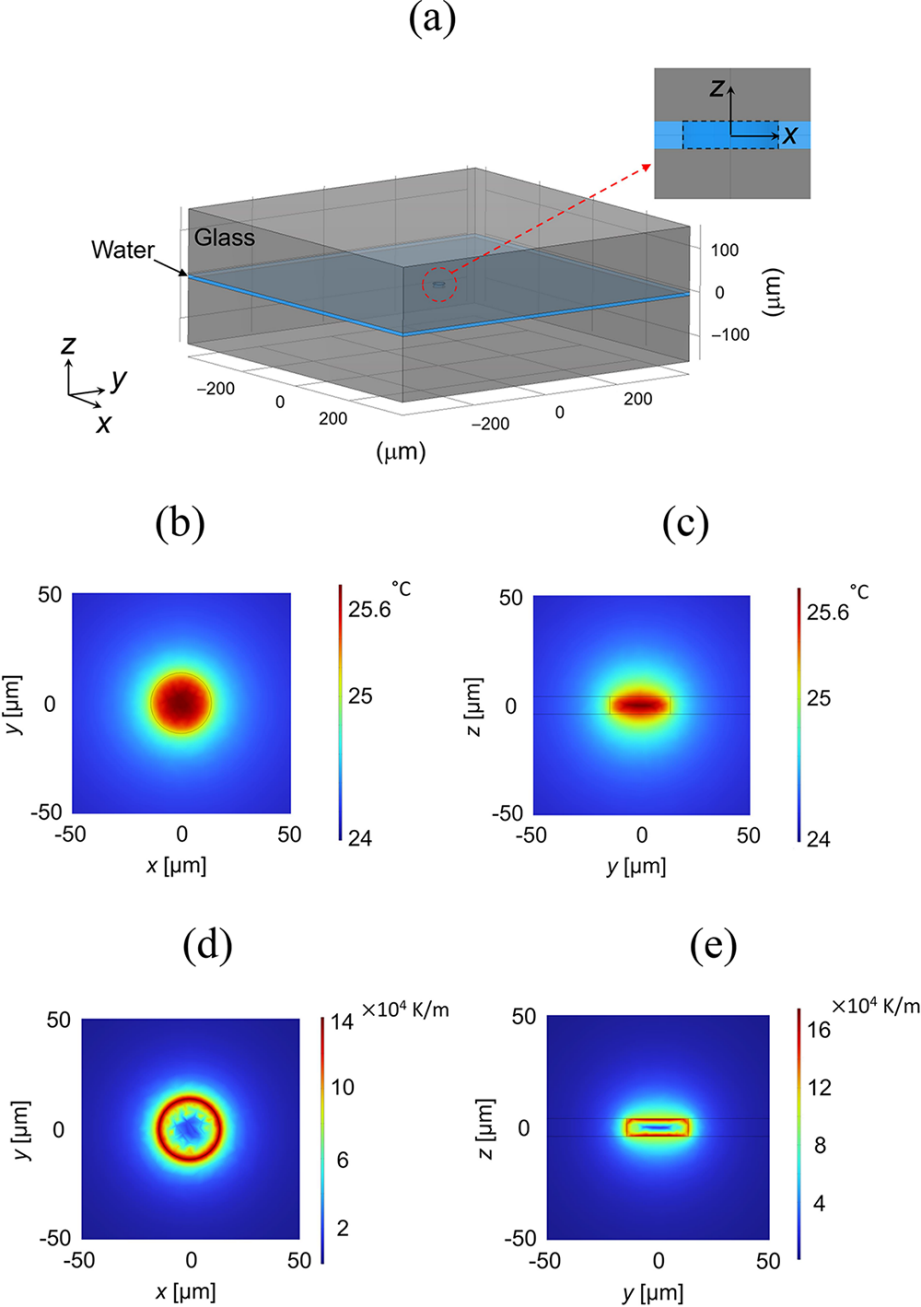
(a) Schematic illustration of the sample chamber, featuring an 8 μm thick water layer sandwiched between 150 μm thick borosilicate glass coverslips, as used in the FEM. (b,c) Temperature distribution calculated for the system shown in (a). (b,c) for *x*-*y* and *y*-*z* planes, respectively. (d,e) Temperature gradient distribution calculated for the system shown in (a). (d) shows the magnitude of temperature gradient in the *x-y* plane, and (e) shows the magnitude of temperature gradient in the *y*–*z* plane.

Here, we consider a scenario where thermophoresis is pronounced: a focused, continuous-wave (CW) laser with a wavelength of 1560 nm is used to heat water. Since the temperature distribution and temperature gradient are in a steady state, the governing equations are the steady-state heat conduction equations of the Poisson and Laplace types, given below:
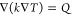
1
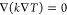
2where *k* is the thermal conductivity of water, *T* is the temperature, *Q* is the heat source. We use eqs 1 and 2 in the domains with and without laser (i.e., heat source), respectively. Next, the system for performing temperature calculations is illustrated in Figure 1a. A 150 μm thick borosilicate glass coverslip sandwiches a water layer with a thickness of 8 μm; this assembly is referred to as the sample chamber. Buoyancy-driven natural convection can interfere with thermophoresis observations when the water layer exceeds about 50 μm.^4,23^ Thermophoresis experiments typically use water layers between 1 and 20 μm.^6,18,24^ The 8 μm thickness in this study was chosen to match the conditions of subsequent experiments. Given the steady-state nature of the temperature field under consideration, the boundary conditions for temperature and heat flux at the interfaces were assumed to be continuous. Furthermore, the boundary conditions for the outer surfaces of the glass substrate and the water were set to room temperature, 24 °C.

The inset in Figure 1a shows a cylindrical heat source placed at the center of the water layer. The dimensions of this heat source were determined based on the parameters of the near-infrared (NIR) laser, as measured in the experiments described later. The diameter of the cylindrical heat source, *d*_pillar_ = 27.6 μm, corresponds to the full width at half-maximum (FWHM) of the intensity profile of the NIR laser focusing spot. For a Gaussian beam, the FWHM in the *z*-direction is estimated to be approximately 140 μm, about five times larger than the FWHM in the *x-y* plane, which is 27.6 μm. Since this value exceeds the 8 μm thickness of the water layer, the entire 8 μm thick water layer is expected to be covered by the Gaussian beam’s *z*-direction FWHM, leading to the installation of a cylindrical heat source in the water. Equation 1 was applied to this water cylinder, with the volumetric heat source *Q*_V_ assigned to *Q* in eq 1. The experimental method for calculating *Q*_V_, taking into account the optical absorption coefficient of water, will be detailed in the experimental section. Consequently, in the subsequent simulation sections, *Q*_V_ will be simply assigned a certain value to compute the temperature field as the output. Equation 2 was applied to the water region without a heat source and the entire glass region with the thermal conductivity of the glass. To account for the temperature dependence of water’s thermal conductivity, the thermal conductivity *k* of water was expressed as *k* = 0.61 + 0.0012(*T* – *T*_0_) W m^–1^ K^–1^, where *T*_0_ = 298 K.^25^ These heat conduction simulations were executed using the FEM solver COMSOL Multiphysics (version 6.2).

Figure 1b,c show the temperature distributions in the *x-y* and *y-z* planes, respectively. In the *x*-*y* plane shown in Figure 1b, the temperature decreases radially from the heat source, which is the laser spot, following a 1/*r* distance dependence.^26^ On the other hand, in the *x*-*y* plane shown in Figure 1c, despite adding a constant amount of heat to the heat source within the cylinder, the temperature decreases within the heat source near the interface between the glass and water in the *z*-direction. This temperature drop is due to the cooling effect of the glass substrate. Figure 1d,e show the magnitude of temperature gradients calculated by differentiating the temperature distributions in Figure 1b,c. As the temperature distribution is almost uniform around the center of the heat source, the temperature gradients are quite small within the heat source. In contrast, near the interface of a cylindrical heat source, the temperature gradient is on the order of 10^5^ K m^–1^. And within a distance of about 50 μm from the interface of the heat source, the temperature gradient is on the order of 10^4^ K m^–1^. These values are sufficient to drive thermophoresis.^4,13^

### 2.2 Brownian Dynamics Simulation

This section describes the numerical methods for Brownian dynamics and the implementation of thermophoretic force. The Langevin equation is typically used as the governing equation to describe Brownian motion. When incorporating the thermophoretic force into the Langevin equation, it can be represented by the following equation
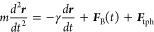
3where *m* is the particle mass, ***r*** is the position of a particle, ***F***_B_ is the random force driving Brownian motion, ***F***_tph_ is the thermophoretic force, and γ is the friction coefficient. Here, since the particle is considered spherical, the friction coefficient is equivalent to drag coefficient obtained from Stokes’ law, which is given by = 6πμ*a*, where μ is the fluid viscosity and *a* is the particle radius. In this simulation, the viscosity μ at each temperature was obtained using the approximation μ = 2.761 exp (1713/*T*)×10^−6^ (Pa s).^27^ Furthermore, since the Reynolds number in the system is sufficiently small (e.g., with kinematic viscosity ν = 1.0 × 10^–6^ (m^2^ s^–1^), particle diameter *d* = 0.5 (μm), and characteristic velocity *v* ≪ 1 (m s^–1^), it follows that Re = *vd*/ν ≪ 1) and the relaxation time of particle is much shorter than the characteristic time scale (e.g., the frame interval of data acquisition in experiments), the system is overdamped, and eq 3 can be rewritten as follows
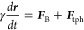
4

This equation is referred to as the overdamped Langevin equation. In this simulation, eq 4 is used as the governing equation for particle motion to obtain numerical solutions for particle behavior.

Next, we consider each driving force in the overdamped Langevin equation. First, we describe the random force ***F***_B_. The random force ***F***_B_ follows a Gaussian distribution in thermal equilibrium, and the displacement distribution due to the random force also follows a Gaussian distribution. Therefore, the random displacement due to Brownian motion Δ***r***^B^ = (Δ*x*^B^, Δ*y*^B^, Δ*z*^B^) over short time step Δ*t* is given by
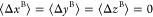
5
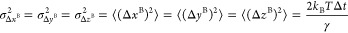
6where , , and  are the variance of displacements in *x, y*, and *z* axis due to Brownian motion, respectively, and *k*_B_ is the Boltzmann’s constant.^28^ From eqs 5 and 6, the random displacement due to Brownian motion is calculated by generating displacements that follow a probability distribution with a mean of zero and a variance of . In the simulations, random numbers following this distribution are generated using the Box-Muller method, and these are used to determine the random displacements. The temperature *T* in the random force is approximated as room temperature *T*_room_. Furthermore, when calculating Brownian motion displacement, same seed values of random numbers are used for every simulation run to focus on the effect other than randomness.

Second, we consider the thermophoretic force. The velocity ***v***_tph_ driven by thermophoresis is known to be given by
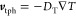
7Here, *D*_T_ represents the thermophoretic mobility.^1^ From this equation, thermophoretic force is expressed by
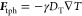
8

The thermophoretic mobility, used in eqs 7 and 8, is known to be temperature-dependent. This temperature dependence of *D*_T_ has been widely reported by researchers such as Braibanti et al. and Tsuji et al.,^29,30^ and its implementation is essential for accurate thermophoretic simulations. Thus, the thermophoretic mobility is represented as *D*_T_(*T*), incorporating the temperature dependence of the thermophoretic mobility in this simulation. In the following simulation sections, we refer to previously reported values by Braibanti et al.,^29^ using linear interpolation to determines the thermophoretic mobility at each position ***r***. In addition, various physical models and measured values for the thermal diffusion coefficient (*D*_T_) have been proposed beyond those mentioned in the referenced literature.^19,31−34^ Since we provide our BDS computational code in Python, one can implement these *D*_T_ models or values within the code. This would allow for comparisons of the diffusive dynamics of thermophoretic behavior across different models.

Then, we perform discretization based on the overdamped Langevin equation obtained from eq 4 and describe its implementation in numerical simulations. Equation 4 can be expressed as follows, where Δ*t* is a time step, *t*_*i*_ = *i*Δ*t* is an accumulated time at the *i*-th step:
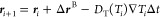
9

Here, ***r***_*i*+1_ is the coordinate of the particle at the *i*+1th step. *T*_*i*_ and ∇*T*_*i*_ are the temperature and temperature gradient at ***r****_i_* given by *T*_*i*_ = *T*(***r****_i_*) and ∇*T*_*i*_ = ∇ *T*|_***r***=***r****_i_*_, respectively.

Finally, we will describe how to implement the BDS in a Python script. In this study, we employ the 2D simulations in the BDS. This approach is justified by the fact that the sample chamber thickness in the FEM simulation is 8 μm, which is sufficiently thin; natural convection effects are negligible and thus a 2D simulation is a valid approximation. Additionally, the fact that differences in particle behavior along the *z*-direction were not observed in experiments described in subsequent sections supports the validity of the 2D simulation. In the 2D simulations, it should be noted that the temperature profiles are computed in 3D in FEM simulation. Therefore, the temperature fields of the 3D-FEM data at *z* = 0, which corresponds to the middle plane of the sample chamber in the direction normal to the glass substrates (see Figure 1a), are used to approximate the 2D temperature profiles in the BDS. In the Python script, the temperature and temperature gradient at arbitrary positions were obtained by first-order interpolation using these 2D temperature profiles.

The simulation is carried out for multiple particles to imitate the real experimental observation and/or to obtain the sufficient number of samples to reduce the effect of noise, while the interparticle interaction is neglected here because the particle concentration is not so high in the experiments.

### 2.3 Numerical Results and Discussion

This section presents the results obtained from the simulations described the above. Using the method outlined in Sections 2.1 and 2.2, we reproduced the temperature field upon laser heating and executed simulations to investigate the diffusion behavior of PSNPs suspended in water. As will be described in the experimental section, laser irradiation in the experiment was maintained for 1200 s to allow the depletion caused by thermophoresis to reach an equilibrium state.^22^ Therefore, in this section, 1000 PSNPs were randomly distributed within a 100 × 100 μm^2^ area at *t* = 0 s, and the diffusion behavior of individual particles was simulated for 1200 s.

**Figure 2:**
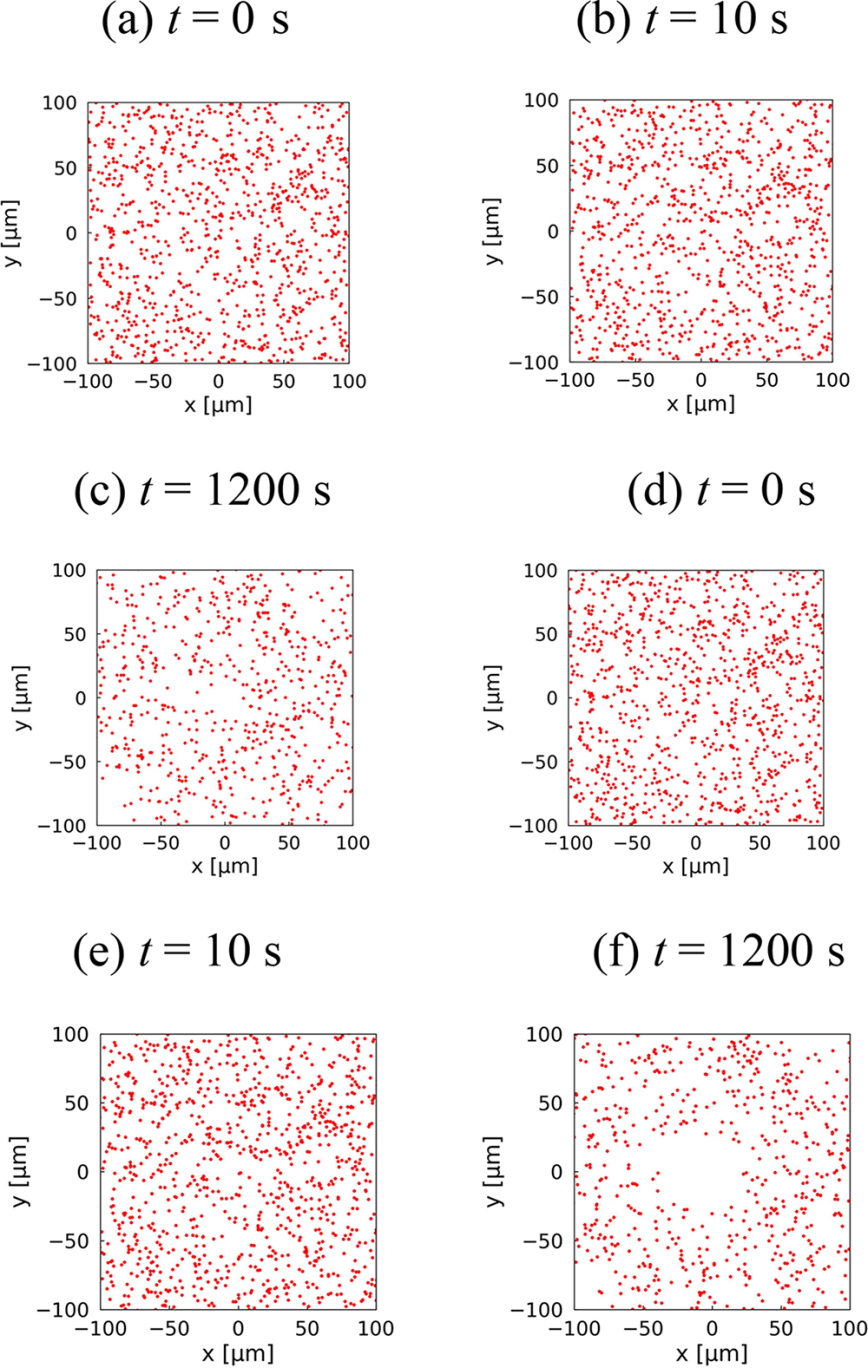
Snapshots of thermophoretic behavior, with PSNPs randomly placed within a 100 × 100 μm^2^ square region. (a) *t* = 0 s, (b) *t* = 10 s, and (c) *t* = 1200 s at a laser power of 20.5 mW (Δ*T*_max_ = 1.77 K). (d) *t* = 0 s, (e) *t* = 10 s, and (f) *t* = 1200 s at a laser power of 79.8 mW (Δ*T*_max_ = 6.88 K).

Here, as representative thermophoretic behavior, the snapshots of the diffusion of individual PSNPs are shown in Figure 2a–c for a laser irradiation power of 20.5 mW (Δ*T*_max_ = 1.77 K) and in Figure 2d–f for a power of 79.8 mW (Δ*T*_max_ = 6.88 K): please note that the Δ*T*_max_ represents the calculated maximum temperature increase in the laser spot. First, examining the snapshots in Figure 2a–c, which correspond to *t* = 0, 10, and 1200 s under the conditions of a small temperature increase, it can be observed that Brownian motion of individual particles is dominant, and the particle distribution appears random. Although in the snapshot of Figure 2c, no particles seem to be present in the central laser spot, no clear depletion pattern was observed, unlike the fluorescence observations at high concentrations.^4,22^ On the other hand, in the case of a larger temperature increase (Δ*T*_max_ = 6.88 K) shown in Figure 2d–f, distinct thermophoretic depletion was observed at 1200 s after a sufficiently long period. Therefore, it can be concluded that the implementation of thermophoretic forces in our BDS model, developed in Section 2.2, is achieved. As will be discussed in detail in the experimental section, the radial concentration calculated from the trajectories of individual PSNPs confirmed that thermophoresis was induced even in the case of a small temperature increase, as shown in Figure 2a–c. Considering previous literature, a small Δ*T*_max_ of about 1 to 2 °C is preferred to eliminate artifacts such as convection and optical trapping,^4,22^ and to suppress nonlinear effects. Therefore, we focus on the data for 20.5 mW (Δ*T*_max_ = 1.77 K) when comparing simulation and experimental results.

## 3 Experiment

### 3.1 Materials and Methods

In the experiments, we used an aqueous dispersion of PSNPs with a diameter of 500 nm that were not surface-modified (Cat# 07307-15, Polysciences, Inc.). These unmodified PSNPs are referred to as bare-PSNP for convenience. The stock solution of the purchased colloids contained a small amount of surfactant for stabilization, which could potentially affect thermophoresis. To address this, the stock solution was centrifuged at 8000 rpm for 3 min, and the solvent was replaced with deionized water. This process was repeated twice to remove the surfactant. The density of bare-PSNP was adjusted with deionized water during this process to achieve a suitable concentration for observation under optical microscope.

The procedure for preparing the sample chamber is as follows. A borosilicate glass coverslip (C024321, Matsunami Glass Ind. LTD) with dimensions of 24 × 32 × 0.15 mm^3^ was used as received. First, a very thin layer of vacuum grease (HIVAC-G, Shin-Etsu Silicone) was applied to the edges of one coverslip. Next, 2 μL of the aforementioned colloidal aqueous solution was placed in the center of the coverslip. Finally, another coverslip was placed on top of the first one, creating a sample chamber with a thin water film sandwiched between the two coverslips. If the application of vacuum grease was insufficient, the colloidal solution may occasionally leak slightly from the chamber. To eliminate the impact of this minor leakage, the sample chamber was left to stand for at least 24 h to ensure there was no reduction in the solution volume before being used for laser irradiation experiments. The thickness of the water layer was approximately 8 μm. This thickness was estimated using a method similar to that reported by the literature,^18^ assuming that the thickness of the glass substrate is 150 μm and that the focusing dial of the microscope moves the objective lens linearly with respect to its rotation.

The optical system used in the experiments is illustrated in Supporting Information Figure S1. An inverted optical microscope (IX-73, Olympus) was used for sample observation and laser irradiation. The PSNPs were observed using a dark-field condenser (U-DCD, Olympus) to capture the scattering images. A monochrome CMOS camera (CS235MU, Thorlabs) was used. For observation and laser irradiation, an objective lens with NA = 0.1 was used (Plan N, Olympus). A CW fiber-output laser with a wavelength of 1560 nm (PUMP-1560, NPI Lasers) served as the laser source. The linearly polarized laser beam was collimated with a plano-convex lens and focused to the diffraction limit by the objective lens before being irradiated onto the sample. The light intensity profile in the *x*-*y* plane at the sample position was measured using a near-infrared camera (Cat# 56-567, 1460–1600 nm Near-Infrared Camera, Edmund Optics), and the beam’s full width half-maximum was found to be 27.6 μm (see Supporting Information Figure S2).

### 3.2 Results and Discussion

We focused the objective lens at a height of *z* = 0 μm in the sample chamber and irradiated the sample with laser powers of 20.5, 40.5, 79.8, and 114 mW. These laser powers were measured by replacing the sample chamber with a power meter. We calculated the temperature distributions and the temperature gradients using these laser powers with the following procedures. The power absorbed by the thin water layer (W) was calculated based on the distance-dependent light intensity relation *I*_out_ = *I*_in_ × exp (−α × *L*). Here, *I*_in_ is the incident laser power (W), *I*_out_ is the transmitted laser power (W), α is the absorption coefficient of water at the laser wavelength of 1560 nm, and *L* is the optical path length of the laser. From the literature, α = 967 (m^–1^) was used,^35^ and the experimental condition gave *L* = 8 μm. The power absorbed by the 8 μm water layer, *I*_abs_, was calculated from the relation *I*_abs_ = *I*_in_ – *I*_out_. As mentioned earlier, the FWHM of the focused laser beam was 27.6 μm, and the water film thickness was 8 μm. Thus, assuming that *I*_abs_ is absorbed by a cylindrical water volume with a diameter of 27.6 μm and height of 8 μm, *I*_abs_ (W) was divided by the volume of this cylinder to obtain the volumetric heat source *Q*_V_ (W m^–3^) used in the temperature calculation described in Section 2.1. The laser powers of 20.5, 40.5, 79.8, and 114 mW corresponded to *Q*_V_ values of 3.3 × 10^10^, 6.5 × 10^10^, 1.3 × 10^11^, and 1.8 × 10^11^ (W m^–3^), respectively. Finally, we obtained the maximum temperature increases (Δ*T*_max_) in the laser spot of 1.77, 3.5, 6.9, and 9.8 °C for the laser powers of 20.5, 40.5, 79.8, and 114 mW, respectively. The method described here, which defines the absorbed heat of the solvent (W) based on the measured laser spot size and estimates the temperature increase due to laser heating using FEM for steady-state heat conduction, has shown good agreement with our previous studies where local temperatures were measured using fluorescence correlation spectroscopy.^26,36^ Therefore, we infer that our temperature estimates are valid. Another commonly used method for evaluating local temperatures involves the temperature dependence of the fluorescence intensity of chromophores.^22^ Since we provide the Python code for the BDS simulation of MST, it is also possible to import two-dimensional temperature distributions obtained through fluorescence microscopy into Python and execute our computational method using those data.

**Figure 3:**
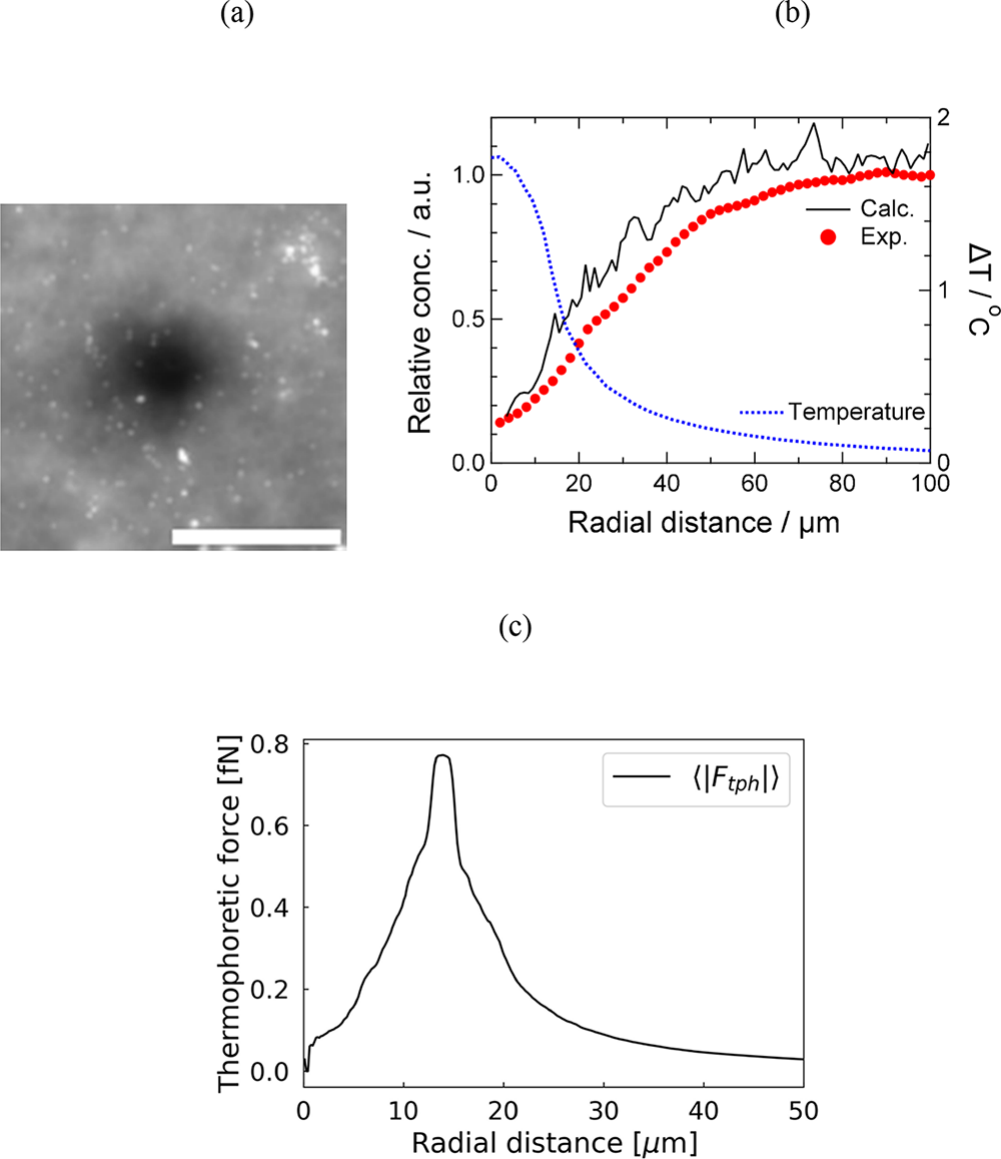
(a) Time-averaged scattering image of the thermophoresis at a laser power of 20.5 mW (Δ*T*_max_ = 1.77 °C). Scale bar: 100 μm. (b) Experimental and calculated radial concentration profiles of PSNPs. Radial temperature profile is also shown for comparison. (c) Calculated thermophoretic force acting on PSNPs as a function of radial distance at a laser power of 20.5 mW (Δ*T*_max_ = 1.77 °C).

Laser irradiation was continued for 1200 s at each laser power while recording video at 1.0 frame per second (FPS) using the CMOS camera. Figure 3a shows an optical micrograph of the scattering intensity averaged from a video taken under conditions of Δ*T*_max_ = 1.77 °C. The temporal averaging was performed over 600 frames from 600 to 1200 s. The video is provided as Supporting Information Video S1. As shown in Video S1, thermophoresis under this irradiation condition begins after several tens of seconds, and the depletion region gradually expands over the course of several hundred seconds. In the time range from 600 to 1200 s, no significant changes in the depletion region were observed. Similar dynamics have been reported in previous experimental and finite element method studies of MST using similar laser spot sizes.^22^ Therefore, we presumed that the averaged scattering image in Figure 3a under the condition of Δ*T*_max_ = 1.77 °C corresponds to the equilibrium state of MST. In Figure 3a, a reduction in scattering intensity can be observed around the laser spot at the center of the image, indicating that the PSNPs have moved away due to thermophoresis. This picture was imaged using an 8-bit CMOS camera, and in regions where no thermophoresis occurred, the time-averaged scattering intensity, after subtracting the background, was approximately 145 counts. Even in the darkest central region of Figure 3a, the scattering intensity remains around 20 counts, indicating that the PSNPs were not completely depleted.

For a more quantitative analysis, sample chambers with different particle concentrations were prepared, and a linear calibration curve of time-averaged scattering intensity under Brownian motion without laser irradiation was created and used to convert Figure 3a into relative particle concentration. The (*r*, θ) coordinate system was set at the center of the image in Figure 3a, and the PSNP concentration profile, averaged in the θ direction, is shown in Figure 3b by the red dotted line. The concentration at the center of the laser spot decreased to approximately 14%, gradually recovering to a steady state at *r* = 60–70 μm. To compare this experimental concentration profile with the BDS simulation results, we calculate the Soret coefficient and thermal diffusion coefficient using a simplified model. For this purpose, the following equation is employed.^4,22^
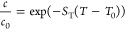
10Here, *c* represents the decreased concentration, *c*_0_ is the initial concentration, *S*_T_ is the Soret coefficient, and (*T* – *T*_0_) is the temperature increase within the laser spot. While the spatial distributions of temperature cannot be ignored in reality, this model allows for a simplified evaluation of the Soret coefficient when thermophoresis is at equilibrium, the temperature increase is minimal, and the contribution of convection is negligible. From Figure 3b, we obtain *c*/*c*_0_ = 0.141, and from the temperature profile in Figure 3b, (*T* – *T*_0_) = 1.77 °C, leading to a calculated *S*_T_ = 1.1 (K^–1^). Given the relation *S*_T_ = *D*_T_/*D*, where *D*_T_ is the thermal diffusion coefficient, and *D*_T_ can be determined if the diffusion coefficient *D* is known. From the Stokes–Einstein relation, the diffusion coefficient of a PSNP with a diameter of 500 nm at 297 K is estimated to be 9.85 × 10^–13^ m^2^/s (e.g., with γ = 0.88 (mPas) obtained from the approximation stated in Section 2.2). Therefore, from Figure 3b, we obtain *D*_T_ = 1.08 × 10^–12^ m^2^ (K^−1^ s^−1^). This value closely matches those reported in the literature for thermophoresis experiments on bare PSNPs of the same particle size at 297 K, which were not surface-modified.^29^ Thus, the comparison between our experimental results in Figure 3 and the BDS simulation results, which use the literature values of *D*_T_(*T*), is expected to be rigorous.

The particle trajectories for Δ*T*_max_ = 1.77 °C, shown in Figure 2a–c, were converted into radial particle concentration using the following procedure. The radial distance from the laser center, ranging from 0 to 100 μm, was divided into 100 bins, and the number of PSNPs in each *i*-th bin, *N*_*i*_^tph^, was counted. Subsequently, under the same conditions, calculations considering only Brownian motion were performed, and the number of PSNPs in each bin, *N*_*i*_^B^, was similarly counted. The relative concentration in the *i*-th bin, defined as *N*_*i*_^tph^/*N*_*i*_^B^, was then used to calculate the density profile of PSNP as a function of the radial coordinate centered on the laser spot. The concentration profile obtained through this procedure is shown by the black solid line in Figure 3b. First, focusing on the central value of the laser spot, the calculated *c*/*c*_0_ is 0.163, which can be considered a satisfactory match with the experimental value of 0.141. Additionally, while there are slight deviations, the behavior of recovery to the steady-state concentration in the radial direction shows good agreement between the experiment and the simulation. Therefore, we conclude that the BDS model for MST developed in this study is valid and successfully reproduces the experimental results. The slight discrepancy in the recovery behavior is likely attributed to the temperature dependence of *D*_T_. In the experiment, we estimated *D*_T_ = 1.08 × 10^–12^ m^2^ (K^−1^ s^−1^) near room temperature, and in the BDS simulation, we used the *D*_T_(*T*) values from the literature by Braibanti et al.,^29^ where the sample is considered equivalent. However, it is known that *S*_T_ and *D*_T_ can exhibit significant variation depending on the heating method and observation system.^37^ Therefore, it is possible that the *D*(*T*) in our system differed slightly from the literature values.

In the BDS calculations, thermophoretic forces acting on the particles can be evaluated. The averaged absolute thermophoretic force ⟨|***F***_tph_|⟩ as a function of radial distance under laser irradiation at a laser power of 20.5 mW (Δ*T*_max_ = 1.77 °C) is shown in Figure 3c. The calculation method of the ⟨|***F***_tph_|⟩ is as follows: First, the thermophoretic behavior under laser irradiation at 20.5 mW (Δ*T*_max_ = 1.77 K) was calculated with 3000 randomly distributed particles within a 50 × 50 μm area at *t* = 0 s. Next, the discretized thermophoretic ***F***_tph*_i*_ was calculated from the eq 8 (i.e., ***F***_tph_*i*_ = γ(*T*_*i*_)*D*_T_(*T*_*i*_)∇*T*_*i*_). Finally, the discretized thermophoretic forces were divided into 100 equal bins within a radius of 50 μm, and the average absolute thermophoretic forces ⟨|***F***_tph_|⟩ were obtained by averaging the absolute values of the forces within each bin. The thermophoretic force profile in Figure 3c naturally follows the temperature profile shown in Figure 3b. In this case, with Δ*T*_max_ = 1.77 °C, the thermophoretic forces are correspondingly weak, with a maximum value of approximately 0.8 fN. From this, the following can be predicted for polystyrene nanoparticles dispersed in water: when irradiating with a near-infrared wavelength laser, by focusing the laser spot to a submicron scale—the current spot diameter being around 30 μm—the thermophoretic force can easily exceed 10 times the present value. Furthermore, considering a temperature increase of up to 100 °C in water, the laser power can be tolerated up to 50 times the current irradiation conditions. Thus, with a focused near-infrared laser, the thermophoretic force is estimated to reach a maximum of approximately subpiconewton levels. On the other hand, using the photothermal conversion of plasmonic nanoparticles could result in temperature gradients an order of magnitude larger. Consequently, much stronger thermophoretic forces may be induced, though limited to the vicinity of the nanoparticle surface. With the BDS simulations for MST developed in this study, it becomes possible to investigate how individual nanoparticles behave in such systems and to design the appropriate temperature distributions through numerical simulation.

Finally, we discuss the laser power dependence of thermophoretic behavior. As mentioned at the beginning of this experimental section, we conducted experiments using four laser powers: 20.5 mW (Δ*T*_max_ = 1.77 °C), 40.5 mW (Δ*T*_max_ = 3.5 °C), 79.8 mW (Δ*T*_max_ = 6.9 °C), and 114 mW (Δ*T*_max_ = 9.8 °C). The radial concentration profiles were calculated using the same procedure as in Figure 3b, and the results for 40.5 mW (Δ*T*_max_ = 3.5 °C) showed satisfactory agreement between experiment and simulation, as presented in Supporting Information Figure S3 (The video of this irradiation condition is shown in Supporting Information Video S2). Although the discrepancy between experiment and simulation is larger in Figure S3 compared to Figure 3b, this can be attributed, as previously mentioned, to differences in *D*_T_ (*T*) at higher temperatures. At higher laser powers, however, as shown in Supporting Information Videos S3 and S4, even after 1200 s of continuous laser irradiation, the thermophoretic depletion region continued to expand and did not reach equilibrium. This is likely due to the convective, despite using a thin sample chamber of only 8 μm. Detailed investigations using FEM simulations have been conducted to study the contribution of convection in thermophoresis under similar laser irradiation conditions.^22^ When combining MST with optical manipulation techniques, it is common to use high-intensity laser beams, and care must be taken during long-term laser irradiation, such as for durations exceeding 1000 s, as the contribution of convection may gradually become more significant.

## 4 Conclusions

In this study, we demonstrated that by generating an arbitrary temperature field using the FEM and incorporating this temperature field into BDSs to account for thermophoresis, we were able to obtain computational results that exhibit satisfactory agreement with experimental validation. Our computational approach is expected to serve as a flexible and effective tool for quantitatively evaluating the thermophoretic behavior of PSNPs in liquid phases at the single-particle level. In this validation, a temperature field was formed by laser-induced single-spot heating, making the thermophoretic behavior relatively straightforward to visualize. However, given that the depletion range of the PSNPs closely matched the experimental results, we believe that the same approach could be effectively applied to more complex temperature fields, such as those involving multiple plasmonic nanoparticles, by using FEM to calculate arbitrary temperature fields and simulating the thermophoretic behavior of nanoparticles. Thus, the findings we obtained not only calculate the behavior of thermophoresis but also provide flexibility in evaluating thermophoresis from various perspectives. This approach is expected to serve as a tool for connecting experimental observations with theoretical predictions and holds promise for further applications in evaluating thermophoresis.

## Supporting Information Available

A COMSOL file and a python code for the BDS (S1), the optical setup (Figure S1), the intensity profile of the focused NIR laser (Figure S2), and the particle concentration as a function of radial distance at a laser power of 40.5 mW (Figure S3) (PDF)
Time evolution of the thermophoresis at laser powers of 20.5 mW (AVI)
Time evolution of the thermophoresis at laser powers of 40.5 mW (AVI)
Time evolution of the thermophoresis at laser powers of 79.8 mW (AVI)
Time evolution of the thermophoresis at laser powers of 114 mW (AVI)

## Supplementary Material

ao4c08170_si_001.pdf

ao4c08170_si_002.avi

ao4c08170_si_003.avi

ao4c08170_si_004.avi

ao4c08170_si_005.avi
