# Supplementary material for: Brownian Dynamics Simulation of Microscale Thermophoresis in Liquid
Source: ACS Omega. 2025 Jan 30;10(5):4526–33. doi: 10.1021/acsomega.4c08170 (PMC11822484; doi:10.1021/acsomega.4c08170)
Supplement: Supplementary file 1 — ao4c08170_si_001.pdf [file ao4c08170_si_001.pdf]

Supporting Information

# Brownian Dynamics Simulation of Microscale Thermophoresis in Liquid

*Koki Ide[1], Tetsuro Tsuji[2], Takayuki Suzuki[3], and Kenji Setoura[*, 4]*

1. Advanced Course of Mechanical System Engineering, Kobe City College of Technology, Kobe, Hyogo 651-2194, Japan.

2. Graduate School of Informatics, Kyoto University, Kyoto 606-8501, Japan.

3. Department of Mechanical Engineering, Kobe City College of Technology, Kobe, Hyogo 651-2194, Japan.

4. Department of Electrical Materials and Engineering, Graduate School of Engineering, University of Hyogo, Himeji, Hyogo, 671-2280, Japan.

**Corresponding Author**

*Email: setoura@eng.u-hyogo.ac.jp

## S1. Codes for numerical simulations.

The COMSOL file for the steady-state heat conduction and the Python code for the Brownian dynamics simulation have been uploaded with the preprint version of this paper. Please download them from the ChemRxiv link below.

https://doi.org/10.26434/chemrxiv-2024-10n14

Figure S1.

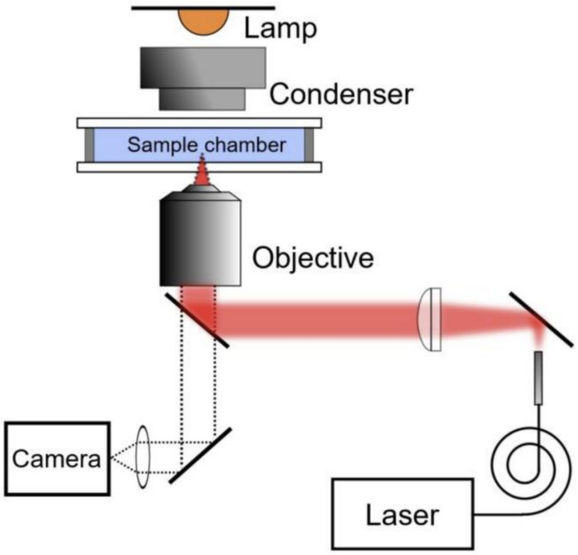


Figure S1. Schematic illustration of an optical setup.

**Figure S2.**

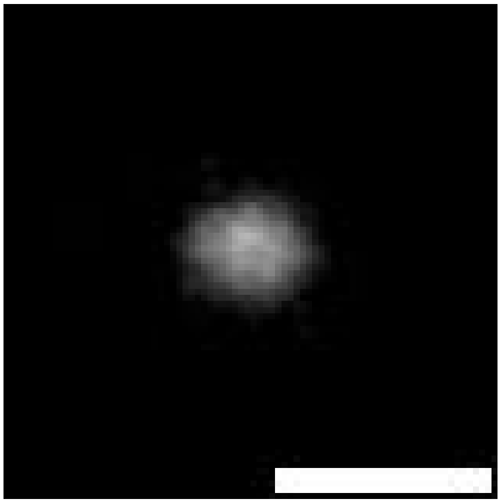

**Figure S2**. Near-infrared (NIR) image of the focused laser at the water-glass interface. Scale bar is 50 μm.

Figure S3.

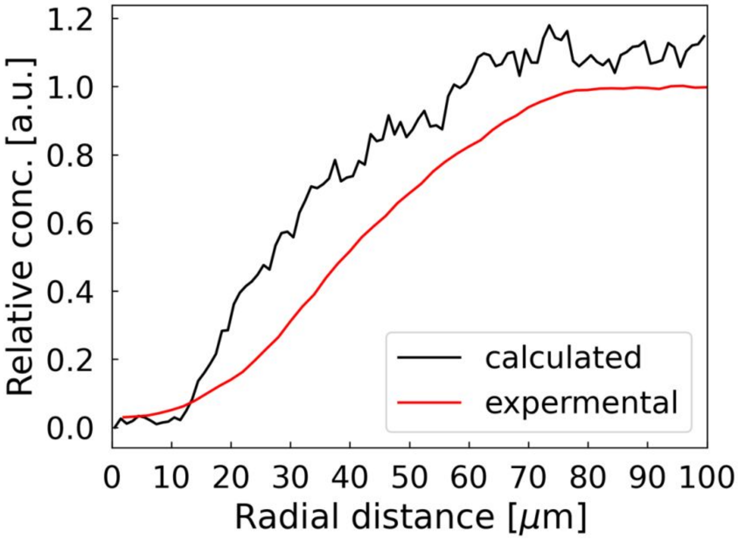


Figure S3. Experimental and calculated radial concentration profiles of PSNPs at a laser power of 40.5 mW ($\Delta T_{max}$ = 3.5 ˚C).
